# Gut microbiome: A potential indicator for predicting treatment outcomes in major depressive disorder

**DOI:** 10.3389/fnins.2022.813075

**Published:** 2022-07-22

**Authors:** Zaiquan Dong, Xiaoling Shen, Yanni Hao, Jin Li, Haizhen Xu, Li Yin, Weihong Kuang

**Affiliations:** ^1^Mental Health Center of West China Hospital, Sichuan University, Chengdu, China; ^2^Department of Psychiatry, National Clinical Research Center for Geriatrics, West China Hospital, Sichuan University, Chengdu, China

**Keywords:** gut microbiota, depression, treatment responses, 16S rRNA sequencing, metabolomics

## Abstract

The therapeutic outcomes in major depressive disorder (MDD), one of the most common and heterogeneous mental illnesses, are affected by factors that remain unclear and often yield unsatisfactory results. Herein, we characterized the composition and metabolic function of the gut microbiota of patients with MDD during antidepressant treatment, based on 16S rRNA sequencing and metabolomics. The microbial signatures at baseline differed significantly between responder and non-responder groups. The gut microbiota of the non-responder group was mainly characterized by increased relative abundances of the phylum Actinobacteria, families Christensenellaceae and Eggerthellaceae, and genera *Adlercreutzia* and *Christensenellaceae R7 group* compared to that of the responder group. Additionally, the gut microbiota composition of the responder and non-responder groups differed significantly before and after treatment, especially at the genus level. Moreover, 20 differential metabolites between the responder and non-responder groups were identified that were mainly involved in lipid metabolism (cholestane steroids and steroid esters). Eggerthellaceae and *Adlercreutzia* displayed strong co-occurrence relationships with certain metabolites, suggesting alternations in the gut microbiome, and associated metabolites may be potential mediators of successful antidepressant treatment. Overall, our study demonstrates that alterations in gut microbiota composition and metabolic function might be relevant to the response to antidepressants, thereby providing insight into mechanisms responsible for their efficacy.

## Introduction

Major depressive disorder (MDD) is one of the most common mental disorders worldwide, occurring with high incidence and causing disability (Kessler et al., 2003; Huang Y. et al., 2019). The main symptoms of MDD include persistent sadness and anhedonia, often accompanied by obvious physical symptoms and insomnia. The lifetime prevalence of MDD in adults is 13–16% (Lépine and Briley, 2011; Greenberg et al., 2015; Johnston et al., 2019). Several methods are currently used to treat patients with MDD. However, despite administration of sufficient doses and maintenance of treatment, 30–40% of patients do not respond to treatment, leading to significant treatment resistance and unsatisfactory results (Maalouf and Brent, 2012; Thomas et al., 2015; Cipriani et al., 2018). Therefore, understanding the mechanism underlying unsatisfactory outcomes of antidepressants is needed to achieve the successful treatment of MDD.

Previous studies have shown that many factors may lead to poor MDD treatment outcomes, including heterogeneity, genetic factors, and individual physiological conditions such as the state of immune homeostasis (Amitai et al., 2016; Manchia et al., 2020). However, these conclusions remain controversial. Thus, exploring factors that affect the effectiveness of MDD treatment continues.

Increasing evidence indicates that the gut microbiota plays a vital role in regulating brain function and human behavior (Mayer et al., 2014; McGuinness et al., 2022). Several observational studies have examined the gut microbiota of patients with MDD (Zheng et al., 2016; Lin et al., 2017; Chen Z. et al., 2018; Cheung et al., 2019; Valles-Colomer et al., 2019; Lai et al., 2021; Chang et al., 2022). In general, higher relative abundances of proinflammatory species such as *Desulfovibrio* and members of family Enterobacteriaceae, and lower relative abundance of short-chain fatty acid producing-bacteria such *Faecalibacterium* are observed in MDD (Simpson et al., 2021). Other studies have reported that the use of probiotics and synbiotics can improve the symptoms of depression or anxiety in patients (Yong et al., 2019; Alli et al., 2022). Additionally, a plant-based diet has been shown to improve depressive symptoms by affecting gut function (Horn et al., 2022). These results indicate that MDD may affect the composition of the gut microbiota; thus, targeting the gut microbiota may provide a new option for its treatment.

In the field of tumor treatment, the gut microbiota can reportedly influence the efficacy of chemotherapy and immunotherapy (Vivarelli et al., 2019). Moreover, the fecal microbiota profile of patients with first-episode psychosis was correlated with their response to antipsychotics (Macedo et al., 2017; Schwarz et al., 2018). Similar findings have been observed in the field of depression treatment. Pisanu and Squassina (2017) reported that altered gut microbiome composition can affect the mechanism of action of antidepressants and regulate their therapeutic effect. Fontana et al. (2020) reported that patients with MDD exhibited obvious gut dysbiosis, and that the gut microbiota characteristics of patients with treatment-resistant MDD differed significantly from those of responders to antidepressants. These results suggest that the gut microbiota is not only related to MDD onset, but also to the effects of antidepressant treatment. Jiang et al. (2015) found that gut microbiota α-diversity was increased in non-responders compared to healthy controls (HCs), but not in responsive patients with MDD. Duan et al. (2021) reported that alterations in gut microbiota composition and metabolic function might be relevant to varying patient responses to the antidepressant escitalopram. Recent animal experiments have also demonstrated that lower relative abundance of Firmicutes and higher relative abundance of Bacteroidetes were associated with the effects of fluoxetine and the tricyclic antidepressant amitriptyline in rats with depression-like behaviors (Zhang et al., 2021). Ye et al. (2021) also reported that vortioxetine hydrobromide may ameliorate depressive symptoms by promoting reconstruction of the gut microbiome. These results further support that the gut microbiota may be an important factor affecting MDD treatment, suggesting broad prospects for enhancing therapeutic success.

The mechanisms underlying the effects of antidepressants on the gut microbiome remain to be fully elucidated. Studies have reported that many antidepressants exert antimicrobial effects (Zhernakova et al., 2016; Macedo et al., 2017), such as selective serotonin reuptake inhibitors (SSRIs) that destroy the bacterial cell wall through efflux inhibition (Munoz-Bellido et al., 2000; Coban et al., 2009; Bohnert et al., 2011). Shen et al. (2021) found that gut microbiota diversity tended to return to the normal state in patients with depression after escitalopram treatment. Further, ketamine can inhibit microorganisms such as *Staphylococcus aureus* and other *Staphylococcus* species (Gocmen et al., 2008; Begec et al., 2013) and has been shown to improve the α- and β-diversity of the gut microbiota (Huang N. et al., 2019). The ketamine enantiomer (R)-ketamine significantly attenuated the relative abundance of members of the phylum Bacteroidetes, genera *Clostridium, Ruminococcus*, and *Butyricimonas*, and class Mollicutes after chronic social defeat stress in susceptible mice (Qu et al., 2017; Yang et al., 2017). These studies suggested that the gut microbiota-brain axis may mediate the antidepressant actions of ketamine and (R)-ketamine. Other studies have shown that the gut microbiota can also influence the therapeutic efficacy of antidepressants by affecting their metabolization (Jourova et al., 2016; Zhang et al., 2018).

In summary, although some interesting findings have been reported, the specific microorganisms or metabolites that impact the efficacy of antidepressants in patients with MDD remain controversial and warrant further research. Limitations of previous studies, such as small sample size, sample heterogeneity, and lack of testing for blood concentration, affected the strength of the results. Therefore, the aim of this study was to further elucidate correlations between the gut microbiota and response to antidepressants in patients with MDD. Additionally, this study included only first-episode and drug-naïve patients to reduce sample heterogeneity and applied 16S rRNA sequencing and metabolomics approaches to improve the robustness of the data.

## Materials and methods

### Research design and participant recruitment

Patients with first-episode MDD and HCs participated in this study. The hospitalized patients with MDD were recruited from the Mental Health Center at West China Hospital, Sichuan University from January to October 2019. All patients were from Chengdu (Sichuan, China), which is a relatively closed area with a unique climate and inhabitants have similar eating habits. Patients with MDD were diagnosed according to the Structured Clinical Interview for DSM-IV (SCID) by two psychiatrists. Patients aged < 18 or > 45 years with organic etiology for their psychiatric symptoms, psychotic features, or intellectual disability were excluded. The HCs included 30 worker volunteers aged 18–45 years. For HCs, diagnosis of mental disorder was excluded by two psychiatrists according to the SCID and 24-item Hamilton Depression Rating Scale (HAMD-24) score was < 7.

To avoid bias caused by the influence of body weight, only subjects with a normal body mass index (BMI, 18.5–22.9) were selected for the study. To further exclude the influence of physical disease, subjects with the following diseases were also excluded from the study: cardiovascular disease (e.g., hypertension); endocrine and metabolic diseases (e.g., diabetes mellitus, obesity, or fatty liver disease); digestive diseases (e.g., liver cirrhosis, irritable bowel syndrome, or inflammatory bowel disease); drug or alcohol abuse; use of antibiotics, probiotics, prebiotics, or symbiotics during the 6 months before collection of fecal samples; known active bacterial, fungal, or viral infections; and obvious dietary preferences (e.g., vegetarians).

All procedures contributing to this study complied with the ethical standards of national and institutional committees on human experimentation and Declaration of Helsinki (1975), as revised in 2008. All procedures involving human participants and patients were approved by the Ethics Committee of West China Hospital (WCH) of Sichuan University (approval number: 2019-268). All participants provided informed consent.

### 24-item Hamilton Depression Rating Scale

The HAMD-24 (Hamilton, 1960) scale was used to assess the severity of depressive symptoms before treatment (baseline) and 8 weeks after treatment. Patients were classified into the responder group if their HAMD-24 score reduction rate (R_HAMD) was ≥ 50%; otherwise, the patient was classified into the non-responder group (Keller, 2003).

### Treatment

All patients received an 8-week intervention with one type of SSRI (citalopram, escitalopram or paroxetine) or serotonin-NE reuptake inhibitor (venlafaxine). The starting dose for 1 week was based on the recommended dosage in the package insert and gradually adjusted to the therapeutic dose. During the study period, patients were not allowed to use drugs other than those prescribed in this study. Prescriptions were provided by psychiatrists and administered by nurses to improve treatment compliance. For patients with insomnia, short-term (not more than 1 month) use of the minimum benzodiazepine dosage was allowed at bedtime.

Antidepressant doses were converted to fluoxetine equivalents using Furukawa’s method, supplemented by the daily defined dose method (Hayasaka et al., 2015).

### Serum collection and analysis of blood drug concentration

After 7 days of continuous medication and at the end of the 8-week period, 3 mL cubital venous blood was collected before medication was administered in the morning. The blood sample was placed in an anticoagulant centrifuge tube containing sodium heparin, centrifuged for 5 min, and the plasma was stored at −80°C for further testing. High-performance liquid chromatography (HPLC) was used to measure the drug concentration in the plasma, as previously described (Olesen and Linnet, 1996).

### Stool collection and polymerase chain reaction amplification

Collected stool samples were quickly frozen at −80°C for further analysis. Microbial DNA was extracted using the QIAamp DNA Stool Mini Kit (Qiagen, Hilden, Germany) and stored at −20°C. The following polymerase chain reaction (PCR) primers were used to amplify the V3–V4 region of the bacterial 16S ribosomal RNA gene: 5′-ACTCCTACGGGAGGCAGCAG-3′ (forward) and 5′-GGACTACHVGGGTWTCTAAT-3′ (reverse). PCR amplification was performed using the TransStart^®^ FastPfu Polymerase system (Transgen Biotech, Beijing, China), including 4 μL 5 × FastPfu Buffer, 2 μL dNTPs (2.5 mM), 0.8 μL each primer (5 μM), 0.4 μL FastPfu Polymerase, and 10 ng template DNA. The reaction mixture was initially denatured at 95°C for 2 min, followed by 25 cycles consisting of 95°C for 30 s, 55°C for 30 s, 72°C for 30 s, and a final extension period of 72°C for 10 min.

### 16S rRNA gene sequencing analysis

Sequencing libraries were constructed according to standard protocol using paired-end sequencing with the Illumina Miseq platform (Illumina Inc., San Diego, CA, United States), as previously described (Bartram et al., 2011). Operational taxonomic units (OTUs) were selected and analyzed using QIIME2 (Kuczynski et al., 2012). The raw reads were deposited in the NCBI Short Read Archive under BioProject ID PRJNA778934.

### Gas chromatography-mass spectrometry analysis

Fecal sample (50 mg) was loaded into a 2 mL centrifuge tube and extracted in 500 μL solution containing 2% L-2 chlorophenylalanine in methyl alcohol:water (4:1) using a 6-mm steel ball. The sample was ground with a frozen tissue grinding machine at −10°C and 50 Hz for 3 min. Then, 200 μL chloroform was added and the sample was ground again at −10°C and 50 Hz for 3 min. Subsequently, an ice-cold mixture of methanol and chloroform was added, the sample was vortexed for 3 min, and then frozen at −20°C for 30 min. The sample was centrifuged at 12,000 rcf for 20 min at 4°C, and the supernatant was placed in a glass derivative flask and dried under vacuum. Following this step, 80 μL methoxypyridine solution (15 mg/mL) was added to the vacuum-dried sample, which was vortexed for 2 min and placed in an oscillating incubator at 37°C for 90 min. BSTFA and n-hexane were added to the mixture, and the samples was derivatized at 70°C for 60 min.

Gas chromatography-mass spectrometry (GC-MS) analysis was performed at room temperature for 30 min using the Agilent 8890 GC system coupled to an Agilent 5977B MSD system (Agilent Technologies, Santa Clara, CA, United States). The temperatures of the MS quadrupole and ion source (electron impact) were set to 150 and 230°C, respectively. The MS data were obtained in full-scan mode (m/z 50–500) and the solvent delay time was set to 5 min. The MS data were analyzed using MassHunter Workstation Quantitative Analysis software (version 10.0.707.0) and metabolites were qualitatively analyzed using the Fiehn database.

### Bioinformatics and statistical analysis

The sequencing data were identified and extracted in FASTQ format using the sequence index files. Barcodes and primers at the beginning and end of the sequences were used to identify and select sequence reads. The sequence number of each sample was normalized, and OTUs with a 97% identity threshold were analyzed using UPARSE software (version 7.1).^1^ Chimeric sequences were identified and removed using UCHIME software (version 4.1).^2^ The taxonomy of each 16S rRNA gene sequence was analyzed by RDP Classifier^3^ using the SILVA 16S rRNA database at a confidence threshold of 70% (Yilmaz et al., 2014).

Ace, Chao1, Simpson, and Shannon indices were used to determine α-diversity. β-diversity was calculated using the “vegan” package (version 2.5-7) in R (version 4.0.5). Bray–Curtis distances were used for principal coordinates analysis (PCoA). Analysis of similarity (ANOSIM) tests were performed to identify differences in β-diversity between groups. Differential key bacteria and metabolites between groups were identified using Welch’s *t*-tests with STAMP analytical software. Only false discovery rate (FDR)-adjusted *p*-values < 0.05 were considered significantly enriched. Linear regression model analysis was performed using the “psych” package (version 2.1.6) in R (version 4.0.5). Correlation analysis between groups was conducted using the Spearman method and the results were displayed as heatmaps and co-occurrence networks.

## Results

### Comparison of clinical and demographic characteristics between patients with major depressive disorder and healthy controls, responders and non-responders

A total of 63 patients (20 males and 43 females) and 30 HCs (10 males and 20 females) were included in this study. Their ages ranged from 19 to 45 years (mean ± SD = 28.34 ± 8.63 years for patients and 29.23 ± 6.59 for HCs). No notable differences were observed in age, sex, marital status, BMI, and history of smoking/drinking both between patients with MDD and HCs (Table 1). No notable differences were observed in age, sex, marital status, family history, BMI, history of smoking/drinking, baseline HAMD/Hamilton Anxiety Rating Scale (HAMA), and fluoxetine equivalent dosage between responders and non-responders (Table 1). The blood drug concentration of all patients was within the reference concentration range recommended by the guidelines (Hiemke et al., 2011). The average dosage and blood drug concentration of antidepressants is shown in Table 2.

**TABLE 1:** Comparison of clinical and demographic characteristics between patients with MDD and HCs, responders, and non-responders.

Group	MDD (*n* = 63)	HCs (*n* = 30)	*p*-value	Responders (*n* = 36)	Non-responders (*n* = 27)	*p*-value
Age (years, mean ± SD)	28.34 ± 8.63	29.23 ± 6.59	0.621	29.86 ± 8.02	26.33 ± 9.14	0.109
Sex, *n* (%)			0.878			
Male	20 (31.7)	10 (33.3)		10 (27.8)	10 (37.0)	
Female	43 (68.3)	20 (66.7)		26 (72.2)	17 (63.0)	
Marital status, *n* (%)			0.954			0.080
Single	29 (46.0)	14 (46.7)		20 (55.6)	9 (33.3)	
Married	34 (54.0)	16 (53.3)		16 (44.4)	18 (66.7)	
Family history, *n* (%)						
Yes	12 (19.0)	*NA*		7 (19.4)	5 (18.5)	0.926
No	51 (81.0)	*NA*		29 (80.6)	26 (81.5)	
BMI (mean ± SD)	21.67 ± 3.91	21.49 ± 2.23	0.815	21.93 ± 3.70	21.33 ± 4.23	0.554
Smoking						0.389
Yes	11 (17.5)	7 (23.3)	0.503	5 (13.9)	6 (22.2)	
No	52 (82.5)	23 (76.7)		31 (86.1)	21 (77.8)	
Drinking						0.195
Yes	22 (34.9)	6 (20.0)		15 (41.7)	7 (25.9)	
No	41 (65.1)	24 (80.0)	0.143	21 (58.3)	20 (74.1)	
HAMD baseline	28.31 ± 7.58	*NA*		26.94 ± 7.73	30.14 ± 7.11	0.097
HAMA baseline	18.75 ± 7.79	*NA*		20.06 ± 8.84	17.00 ± 5.84	0.125
Antidepressant^#^ (mg/day)	42.33 ± 11.88	*NA*		43.04 ± 11.15	41.39 ± 12.94	0.589

HAMD, Hamilton Depression Rating Scale; HAMA, Hamilton Anxiety Rating Scale; NA, not applicable; MDD, major depressive disorder; HCs, healthy controls; SD, standard deviation; BMI, body mass index.

^#^Fluoxetine-equivalent dose of antidepressant medication.

**TABLE 2:** Average dosage and blood drug concentration of antidepressants.

Antidepressant	Dose (mg, mean ± SD)	Blood drug concentration (ng/mL, mean ± SD)
Citalopram (*n* = 19)	34.74 ± 6.12	85.71 ± 17.54
Escitalopram (*n* = 16)	16.25 ± 3.42	48.04 ± 21.67
Paroxetine (*n* = 8)	38.75 ± 6.41	68.48 ± 23.20
Venlafaxine (*n* = 20)	198.75 ± 44.04	235.48 ± 70.97

### Differences in gut microbiota composition between patients with major depressive disorder and healthy controls

**FIGURE 1:**
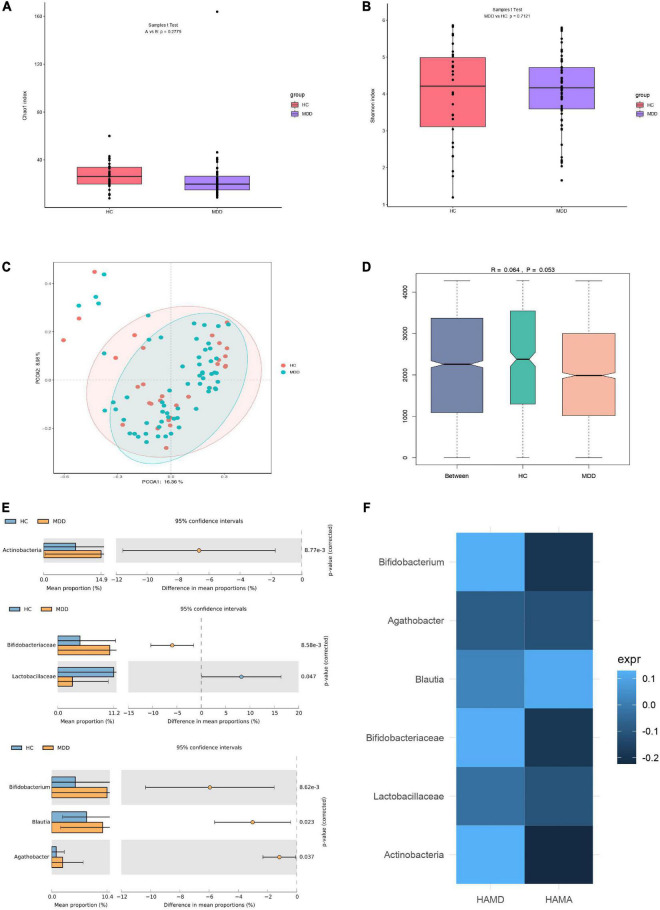
Differences in gut microbiota between MDD patients and HCs. **(A)** Bacterial richness estimated using the Chao1 index. **(B)** Bacterial diversity estimated using the Shannon index. **(C,D)** β-diversity of gut microbiota according to principal coordinate analysis (PCoA) and analysis of similarities (ANOSIM) based on Bray–Curtis distances. **(E)** Significantly different species according to FDR-adjusted *p*-values, calculated using Welch’s *t*-test. The mean abundance of the blue (yellow) community differed between MDD patients and HCs. **(F)** Heatmap of correlations between significantly different species and clinical indicators, determined using the Spearman method.

A total of 63 and 30 stool samples were collected from patients with MDD and HCs, respectively. Accounting for 70% of the valid sequences, 3,202,267 and 1,653,393 high-quality sequences were obtained from patients with MDD and HCs, respectively. After rarefaction of the samples to equal sequencing depth (52,211 reads per sample) and clustering, 4,855,660 sequences from 93 fecal samples were grouped into 1,249 OTUs for downstream analysis. Statistical analysis of α-diversity at the OTU level revealed that the Chao1 and Shannon indices of the microbial communities did not differ significantly between patients with MDD and HCs (Figures 1A,B). To evaluate differences in microbial β-diversity, PCoA and ANOSIM were conducted based on Bray–Curtis distances. Samples from patients with MDD and HCs were clustered into indiscriminate groups based on β-diversity estimates, as assessed by ANOSIM tests (*R* = 0.064, *p* = 0.053, Figures 1C,D). These results indicated that the gut microbiota of patients with MDD was not significantly different from that of HCs.

However, evaluating the gut microbiome composition revealed that patients with MDD and HCs exhibited significant differences at the phylum, family, and genus levels. The relative abundances of the phylum Actinobacteria, family Bifidobacteriaceae, and genera *Bifidobacterium*, *Blautia*, and *Agathobacter* were significantly higher in the microbiota of patients with MDD than in that of HCs. However, the relative abundance of the family Lactobacillaceae was significantly lower in the microbiota of patients with MDD than in that of HCs (Figure 1E). Moreover, the relationship between these differential species and the severity of anxiety and depression was investigated. No significant correlation was detected between species and HAMD or HAMA (Figure 1F).

### Gut microflora differed between responder and non-responder groups at baseline

**FIGURE 2:**
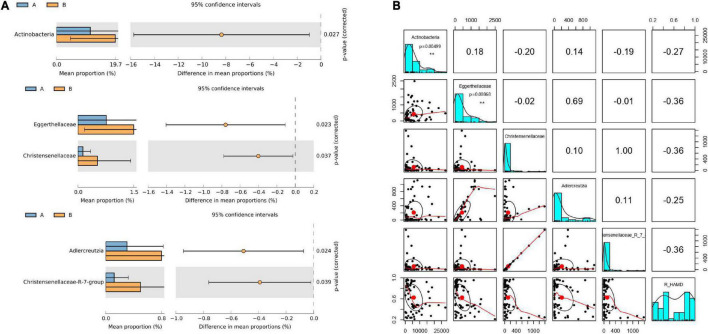
Gut microflora at baseline differed between responder and non-responder groups. **(A)** Significantly different species according to FDR-adjusted *p*-values, calculated using Welch’s *t*-test. The mean abundance in the blue (yellow) community differed between responder and non-responder groups. **(B)** Correlation of R_HAMD with differential species (*R*^2^ = 0.3256), negative correlation with Actinobacteria (*p* = 0.00499), Eggerthellaceae (*p* = 0.00868), *Christensenellaceae*, *Adlercreutzia*, and *Christensenellaceae R7 group*. **p < 0.01.

The 63 stool samples from patients with MDD were divided into responder and non-responder groups, classified based on R_HAMD values. The gut microbiota of the responder and non-responder groups exhibited significant differences at the phylum, family, genus, and OTU levels. The relative abundances of the phylum Actinobacteria, families Christensenellaceae and Eggerthellaceae, and genera *Adlercreutzia* and *Christensenellaceae R7 group* were significantly lower in the responder group than in the non-responder group (Figure 2A).

To further determine whether alterations in the gut microbiota directly contribute to R_HAMD, linear regression model analysis was performed. The results revealed that R_HAMD was correlated with differential species (*R*^2^ = 0.3256) and negatively correlated with Actinobacteria (*p* = 0.00499), Eggerthellaceae (*p* = 0.00868), Christensenellaceae, *Adlercreutzia*, and *Christensenellaceae R7 group* (Figure 2B). These results indicated that the gut microflora significantly affected R_HAMD, especially Actinobacteria and Eggerthellaceae, which were significantly negatively correlated with R_HAMD.

### Metabolic signatures differed between responder and non-responder groups at baseline

**FIGURE 3:**
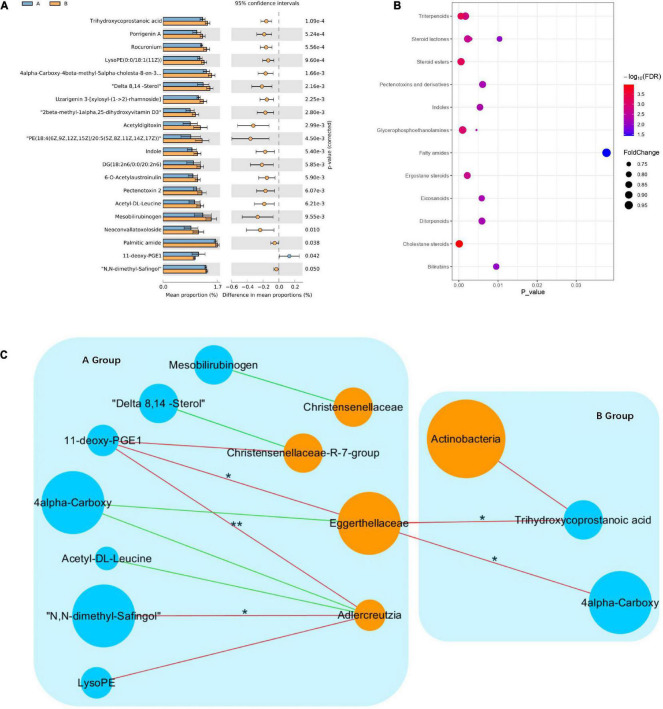
Changes in metabolic signatures between responder and non-responder groups at baseline. **(A)** Histogram based on differentially abundant metabolites between the responder and non-responder groups, determined by Welch’s *t*-test. **(B)** Pathway enrichment analysis of differentially abundant metabolites. Lipid metabolism is the predominant metabolic pathway. **(C)** Interaction networks among differential species and metabolites; node size indicates abundance, different colors represent metabolites (blue) and species (orange), negative correlations are shown as red lines, positive correlations are shown as green lines (Spearman correlations, *p < 0.05, **p < 0.01).

To explore the effects of drug therapy on the metabolic pathways of the gut microbiome, GC-MS-based metabolomics was used to compare the metabolic characteristics of the responder and non-responder groups at baseline. In total, the expression of 20 metabolites differed significantly between the responder and non-responder groups at baseline (*p* < 0.05) (Figure 3A). Specifically, the metabolites enriched in the responder group included trihydroxycoprostanoic acid, diacylglycerols, 4alpha-carboxy, 11-deoxy-prostaglandin (PG) E1, delta 8,14-sterol, porrigenin A, N,N-dimethyl-safingol, mesobilirubinogen, indole, and acetyl-DL-leucine. Meanwhile, the metabolites enriched in the non-responder group included 4alpha-carboxy, 6-O-acetylaustroinulin, 11-deoxy-PGE1, delta 8,14-sterol, porrigenin A, N,N-dimethyl-safingol, mesobilirubinogen, acetyl-DL-leucine, uzarigenin-3, acetyldigitoxin, and phosphatidylethanolamine (PE) (Figure 3B). These differential metabolites were mainly involved in lipid metabolism (cholestane steroids and steroid esters).

To investigate potential interactions between altered gut bacteria and metabolites, a co-occurrence network was constructed based on the results of the Spearman correlation analysis. In the responder group, the genus *Adlercreutzia* was significantly negatively correlated with N,N-dimethyl-safingol and 11-deoxy-PGE1, while the family Eggerthellaceae was significantly negatively correlated with 11-deoxy-PGE1. In the non-responder group, the family Eggerthellaceae was significantly negatively correlated with trihydroxycoprostanoic acid and 4alpha-carboxy. These findings indicated that significant differences in the metabolic pathways in the responder and non-responder groups at baseline were caused by different species in the gut microbiota (Figure 3C).

### Dynamic changes in the gut microbiota of patients with major depressive disorder before and after treatment

**FIGURE 4:**
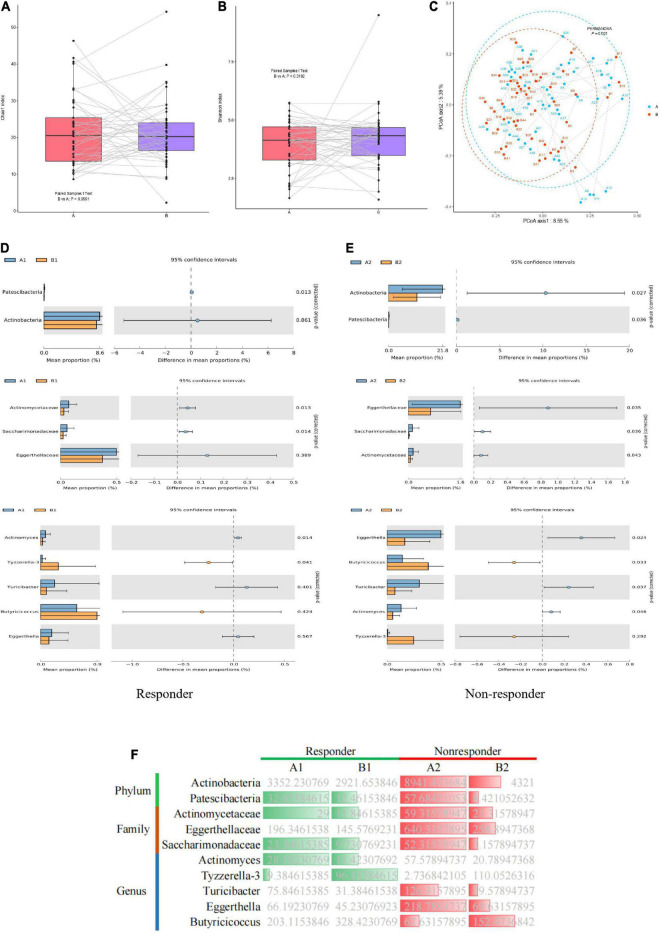
Dynamic changes in gut microbiota of MDD patients before and after treatment. **(A)** Bacterial richness estimated using the Chao1 index. **(B)** Bacterial diversity estimated using the Shannon index. **(C)** β-diversity determined *via* PERMANOVA based on Bray–Curtis distances. Paired samples from the same individual before and after treatment are represented by dotted lines. **(D,E)** Significantly different species according to FDR-adjusted *p*-values, calculated using Welch’s *t*-test. The mean abundance in the blue (yellow) community differed before and after treatment. **(F)** Heatmap showing significantly different microflora before and after treatment in the responder and non-responder groups. Green indicates a significant difference in species abundance in the responder group before and after treatment. Red indicates a significant difference in species abundance in the non-responder group before and after treatment.

Among the 63 samples from patients with MDD, 45 samples (26 in the responder group and 19 in the non-responder group) were collected after 8 weeks of treatment. Changes in the gut microflora before and after treatment were investigated to assess the effect of antidepressant treatment on microbial diversity. Microbial richness, including the Chao1 and Shannon indices, did not differ significantly before and after treatment (Figures 4A,B). However, samples from the responder and non-responder groups were clustered into discriminate groups based on β-diversity estimates, as assessed by PERMANOVA tests (*p* = 0.001, Figure 4C). The results indicated that the distance between samples was significantly reduced after antidepressant treatment (*p* = 0.001), and differences between paired samples from the same individual were significant.

In the responder group, the relative abundances of the phylum Patescibacteria, families Actinomycetaceae and Saccharimonadaceae, and genus *Actinomyces* in the gut microbiota were significantly higher before treatment than after treatment. However, the relative abundance of the genus *Tyzzerella-3* in the gut microbiota of the responder group was significantly lower before treatment than after treatment (Figure 4D). In the non-responder group, the relative abundances of the phyla Actinobacteria and Patescibacteria, families Eggerthellaceae and Saccharimonadaceae, and genera *Eggerthella*, and *Turicibacter* in the gut microbiota were significantly higher before treatment than after treatment. However, the relative abundance of the genus *Butyricicoccus* in the microbiota of the non-responder group was significantly lower before treatment than after treatment (Figure 4E). Thus, significant differences were observed in the gut microbiota of the responder and non-responder groups before and after treatment. Furthermore, dynamic changes in species were observed before and after treatment between the responder and non-responder groups, especially at the genus level (Figure 4F).

## Discussion

Extensive efforts have been made to elucidate the pathogenesis of MDD and predict the efficacy of its treatment (Maes et al., 1997). To this end, this study searched for possible targets in the gut microbiota. No significant differences in richness and diversity were observed between the gut microbiota of patients with MDD and HCs, which has been confirmed in many studies, including our previous research (Vinberg et al., 2019; Mason et al., 2020; Dong et al., 2021). However, other studies have found significant differences in the richness or diversity of the microbiota between patients with MDD and normal controls, such as decreased α-diversity in patients with MDD (Kelly et al., 2016; Liu et al., 2016; Huang et al., 2018; Chung et al., 2019; Sanada et al., 2020; Ye et al., 2021).

The taxonomic findings of the current study indicated that the relative abundances of the phylum Actinobacteria, family Bifidobacteriaceae, and genera *Bifidobacterium*, *Blautia*, and *Agathobacter* were significantly higher in the gut microbiota of patients with MDD than in that of HCs. However, the relative abundance of the family Lactobacillaceae was significantly lower in the gut microbiota of patients with MDD than in that of HCs. Chen J. J. et al. (2018) and Lai et al. (2021) also reported higher relative abundance of the phylum Actinobacteria in patients with MDD than in HCs. However, the study by Chen J. J. et al. (2018) found that the higher relative abundance of Actinobacteria was positively correlated with symptoms of MDD, which is inconsistent with the results of the present study.

Conflicting results have been reported for the relative abundance of the family Bifidobacteriaceae in the gut microbiota of patients with MDD. Several studies have found a higher relative abundance of Bifidobacteriaceae in patients with MDD than in HCs (Chen J. J. et al., 2018; Rong et al., 2019; Lai et al., 2021), which is consistent with the results of the present study. Meanwhile, Jackson et al. (2018) and Aizawa et al. (2016) did not reach this conclusion. The reason for the discrepancy may be that the current study selected patients with first-onset depression, most of whom were in the early stages of the disease. In an animal study conducted by Klünemann et al. (2021), stress caused an increase in the relative abundance of the genus *Bifidobacterium* and supplementation with *Bifidobacterium* was suggested to prevent the onset of depression from stress. These results suggest that the increased relative abundance of *Bifidobacterium* spp. in patients with MDD in the early stage of the disease may be based on a protective mechanism.

Additionally, the relative abundance of the genus *Blautia* was higher in the gut microbiota of patients with MDD than in that of HCs, which aligns with some previous reports (Zheng et al., 2016; Chen J. J. et al., 2018; Chung et al., 2019), but not all studies (Liu et al., 2016; Huang et al., 2018). Moreover, the relative abundance of the family Lactobacillaceae was reduced in the gut microbiota of patients with MDD compared to that of HCs. Aizawa et al. (2016) found that the relative abundance of *Lactobacillus* was reduced in patients with MDD compared to HCs, which verified our conclusion to a certain extent. However, the association between the genus *Agathobacter* and depression has not previously been reported.

This study focused on exploring the relationship between changes in the gut microflora and drug efficacy in patients with MDD treated with antidepressants. The results showed that the gut microbiota of the responder and non-responder groups exhibited significant differences in terms of both their microbial composition and metabolic pathways. The relative abundances of the phylum Actinobacteria, families Christensenellaceae and Eggerthellaceae, and genera *Adlercreutzia* and *Christensenellaceae R7 group* were significantly lower in the responder group than in the non-responder group. Among them, Actinobacteria and Eggerthellaceae were associated with R_HMAD. Previous research has demonstrated that the gut microbiota may also affect the metabolism of antidepressants. Prolonged or high consumption of drugs leads to the accumulation of drug ingredients in intestinal bacteria, thereby weakening the effect of the drug (Klünemann et al., 2021). In addition, this accumulation may also change the metabolism of the gut microbiota, further affecting the effectiveness of the drug and even causing side effects (Klünemann et al., 2021). Siopi et al. (2020) reported that changes in the gut microbiota can minimize the efficacy of SSRIs *via* alterations in the serotonergic pathway of tryptophan metabolism. This result implies that the baseline composition of the gut bacterial community may decrease the efficacy of certain drugs, which could help to predict treatment outcomes. Traditional pharmacology and toxicology should therefore consider the influence of gut bacteria on drug efficacy when evaluating candidate drugs (Long-Smith et al., 2020).

The responder and non-responded groups also exhibited significant differences in lipid metabolism, which was affected by differential bacterial species between the two groups. For example, the abundance of the family Eggerthellaceae was significantly negatively correlated with 11-deoxy-PGE1 in the responder group. Zhao et al. (2014) reported that the abundance of Eggerthellaceae affected lipid metabolism in patients with radiation enteritis, which is consistent with the results of the current study. The lipid metabolic function of the gut microbiota can affect the production of short-chain fatty acids and further affect host systemic inflammation (Furusawa et al., 2013). Yamamura et al. (2021) reported that elevated lipid metabolism and its end-products can help to maintain the integrity of the intestinal barrier and influence the therapeutic effect of probiotics on anxiety and depressive symptoms. Taken together, these findings indicate that the gut microflora may regulate lipid metabolism by reducing the abundance of some bacteria, which affects drug efficacy. The effect of antidepressants on MDD may require sufficient metabolic function of the gut microbiota at baseline.

In addition, the gut microbiome of the responder and non-responder groups displayed different dynamic changes after 8 weeks of drug treatment, especially at the genus level. This finding may reflect interaction between the drug and gut bacteria. Thus, not only will the gut bacteria affect the metabolism and therapeutic efficacy of the drug, but the drug may also affect the composition of the gut bacterial community. Notably, some drugs exert an antimicrobial effect (Ye et al., 2021). For example, tricyclic antidepressants show antiplasmid activity, while SSRIs are active against gram-positive bacteria (e.g., *Staphylococcus* and *Enterococcus*) and Enterobacteria (Zhang et al., 2021). Moreover, paroxetine can reportedly increase the abundance of *Eubacterium ramulus* (Vich Vila et al., 2020). A recent systematic metagenomic analysis demonstrated that both tricyclic antidepressants and SSRIs were associated with interindividual variation of the gut microbiota (Duan et al., 2021). However, the mechanisms underlying the antimicrobial actions of these drugs and the extent to which these effects could play a role in their therapeutic efficacy remain unknown.

This study has a few limitations. First, 16S rRNA gene sequencing has some shortcomings relative to shotgun metagenomic sequencing for functional gene analysis of the gut microbiota, which may weaken the robustness of the results. Second, the sample size in our study was relatively small; thus, a larger population is needed to verify the study findings. Finally, information on dietary habits was not collected.

In summary, the baseline characteristics of the gut microbiome and its metabolism are related to the treatment outcomes of MDD. Thus, assessment of the baseline gut microbiota may help to predict and improve the effects of antidepressants in patients with MDD. The results of this study provide a new approach for maximizing the therapeutic effects of antidepressants and personalized MDD treatment, and may aid in promoting the development of products that improve the function of the gut microbiota.

## Data availability statement

The original contributions presented in the study are publicly available. This data can be found here:NCBI Short Read Archive https://www.ncbi.nlm.nih.gov/sra under BioProject ID PRJNA778934.

## Ethics statement

The studies involving human participants were reviewed and approved by the Ethics Committee of West China Hospital of Sichuan University. The patients/participants provided their written informed consent to participate in this study.

## Author contributions

ZD and WK: conception of the work, final approval of the version to be published, and agreement to be accountable for all aspects of the work. ZD, XS, YH, JL, HX, and LY: acquisition of data. ZD and LY: analysis and interpretation of data for the work. ZD: writing. WK: revision of the work. All authors contributed to the article and approved the submitted version.

## Conflict of interest

The authors declare that the research was conducted in the absence of any commercial or financial relationships that could be construed as a potential conflict of interest.

## Publisher’s note

All claims expressed in this article are solely those of the authors and do not necessarily represent those of their affiliated organizations, or those of the publisher, the editors and the reviewers. Any product that may be evaluated in this article, or claim that may be made by its manufacturer, is not guaranteed or endorsed by the publisher.

## Footnotes

1
http://drive5.com/uparse/

2
http://drive5.com/uchime/

3
http://rdp.cme.msu.edu/

## References

[B1] Aizawa E. Tsuji H. Asahara T. Takahashi T. Teraishi T. Yoshida S. (2016). Possible association of *Bifidobacterium* and *Lactobacillus* in the gut microbiota of patients with major depressive disorder. *J. Affect. Disord.* 202 254–257. 10.1016/j.jad.2016.05.038 27288567

[B2] Alli S. R. Gorbovskaya I. Liu J. C. W. Kolla N. J. Brown L. Müller D. J. (2022). The gut microbiome in depression and potential benefit of prebiotics, probiotics and synbiotics: a systematic review of clinical trials and observational studies. *Int. J. Mol. Sci.* 23:4494. 10.3390/ijms23094494 35562885 PMC9101152

[B3] Amitai M. Taler M. Carmel M. Michaelovsky E. Eilat T. Yablonski M. (2016). The relationship between plasma cytokine levels and response to selective serotonin reuptake inhibitor treatment in children and adolescents with depression and/or anxiety disorders. *J. Child Adolesc. Psychopharmacol.* 26 727–732. 10.1089/cap.2015.0147 26771135

[B4] Bartram A. K. Lynch M. D. J. Stearns J. C. Moreno-Hagelsieb G. Neufeld J. D. (2011). Generation of multimillion-sequence 16S rRNA gene libraries from complex microbial communities by assembling paired-end Illumina reads. *Appl. Environ. Microbiol.* 77 3846–3852. 10.1128/AEM.02772-10 21460107 PMC3127616

[B5] Begec Z. Yucel A. Yakupogullari Y. Erdogan M. A. Duman Y. Durmus M. (2013). The antimicrobial effects of ketamine combined with propofol: an in vitro study. *Braz. J. Anesthesiol.* 63 461–465. 10.1016/j.bjane.2012.09.004 24565343

[B6] Bohnert J. A. Szymaniak-Vits M. Schuster S. Kern W. V. (2011). Efflux inhibition by selective serotonin reuptake inhibitors in *Escherichia coli*. *J. Antimicrob. Chemother.* 66 2057–2060. 10.1093/jac/dkr258 21700628

[B7] Chang L. Wei Y. Hashimoto K. (2022). Brain–gut–microbiota axis in depression: a historical overview and future directions. *Brain Res. Bull.* 182 44–56. 10.1016/j.brainresbull.2022.02.004 35151796

[B8] Chen J. J. Zheng P. Liu Y. Y. Zhong X. G. Wang H. Y. Guo Y. J. (2018). Sex differences in gut microbiota in patients with major depressive disorder. *Neuropsychiatr. Dis. Treat.* 14 647–655. 10.2147/NDT.S159322 29520144 PMC5833751

[B9] Chen Z. Li J. Gui S. Zhou C. Chen J. Yang C. (2018). Comparative metaproteomics analysis shows altered fecal microbiota signatures in patients with major depressive disorder. *Neuroreport* 29 417–425. 10.1097/WNR.0000000000000985 29432299

[B10] Cheung S. G. Goldenthal A. R. Uhlemann A. C. Mann J. J. Miller J. M. Sublette M. E. (2019). Systematic review of gut microbiota and major depression. *Front. Psychiatry* 10:34. 10.3389/fpsyt.2019.00034 30804820 PMC6378305

[B11] Chung Y. E. Chen H. C. Chou H. L. Chen I. M. Lee M. S. Chuang L. C. (2019). Exploration of microbiota targets for major depressive disorder and mood related traits. *J. Psychiatr. Res.* 111 74–82. 10.1016/j.jpsychires.2019.01.016 30685565

[B12] Cipriani A. Furukawa T. A. Salanti G. Chaimani A. Atkinson L. Z. Ogawa Y. (2018). Comparative efficacy and acceptability of 21 antidepressant drugs for the acute treatment of adults with major depressive disorder: a systematic review and network meta-analysis. *Lancet* 391 1357–1366. 10.1016/S0140-6736(17)32802-7 29477251 PMC5889788

[B13] Coban A. Y. Cayci Y. T. Uludağ S. K. Durupinar B. (2009). Investigation of antibacterial activity of sertralin. *Mikrobiyol. Bul.* 43 651–656. 20084919

[B14] Dong Z. Shen X. Hao Y. Li J. Li H. Xu H. (2021). Gut microbiome: a potential indicator for differential diagnosis of major depressive disorder and general anxiety disorder. *Front. Psychiatry* 12:651536. 10.3389/fpsyt.2021.651536 34589003 PMC8473618

[B15] Duan J. Huang Y. Tan X. Chai T. Wu J. Zhang H. (2021). Characterization of gut microbiome in mice model of depression with divergent response to escitalopram treatment. *Transl. Psychiatry* 11:303. 10.1038/s41398-021-01428-1 34016954 PMC8138009

[B16] Fontana A. Manchia M. Panebianco C. Paribello P. Arzedi C. Cossu E. (2020). Exploring the role of gut microbiota in major depressive disorder and in treatment resistance to antidepressants. *Biomedicines* 8:311. 10.3390/biomedicines8090311 32867257 PMC7554953

[B17] Furusawa Y. Obata Y. Fukuda S. Endo T. A. Nakato G. Takahashi D. (2013). Commensal microbe-derived butyrate induces the differentiation of colonic regulatory T cells. *Nature* 504 446–450. 10.1038/nature12721 24226770

[B18] Gocmen S. Buyukkocak U. Caglayan O. (2008). In vitro investigation of the antibacterial effect of ketamine. *Ups. J. Med. Sci.* 113 39–46. 10.3109/2000-1967-211 18521797

[B19] Greenberg P. E. Fournier A. A. Sisitsky T. Pike C. T. Kessler R. C. (2015). The economic burden of adults with major depressive disorder in the United States (2005 and 2010). *J. Clin. Psychiatry* 76 155–162. 10.4088/JCP.14m09298 25742202

[B20] Hamilton M. (1960). A rating scale for depression. *J. Neurol. Neurosurg. Psychiatry* 23 56–62. 14399272 10.1136/jnnp.23.1.56 PMC495331

[B21] Hayasaka Y. Purgato M. Magni L. R. Ogawa Y. Takeshima N. Cipriani A. (2015). Dose equivalents of antidepressants: evidence-based recommendations from randomized controlled trials. *J. Affect. Disord.* 180 179–184. 10.1016/j.jad.2015.03.021 25911132

[B22] Hiemke C. Baumann P. Bergemann N. Conca A. Dietmaier O. Egberts K. (2011). . AGNP consensus guidelines for therapeutic drug monitoring in psychiatry: update 2011. *Pharmacopsychiatry* 44 195–235. 10.1055/s-0031-1286287 21969060

[B23] Horn J. Mayer D. E. Chen S. Mayer E. A. (2022). Role of diet and its effects on the gut microbiome in the pathophysiology of mental disorders. *Transl. Psychiatry* 12:164. 10.1038/s41398-022-01922-0 35443740 PMC9021202

[B24] Huang Y. Shi X. Li Z. Shen Y. Shi X. Wang L. (2018). Possible association of Firmicutes in the gut microbiota of patients with major depressive disorder. *Neuropsychiatr. Dis. Treat.* 14 3329–3337. 10.2147/NDT.S188340 30584306 PMC6284853

[B25] Huang N. Hua D. Zhan G. Li S. Zhu B. Jiang R. (2019). Role of actinobacteria and coriobacteria in the antidepressant effects of ketamine in an inflammation model of depression. *Pharmacol. Biochem. Behav.* 176 93–100. 10.1016/j.pbb.2018.12.001 30528936

[B26] Huang Y. Wang Y. Wang H. Liu Z. Yu X. Yan J. (2019). Prevalence of mental disorders in China: a cross-sectional epidemiological study. *Lancet Psychiatry* 6 211–224. 10.1016/S2215-0366(18)30511-X 30792114

[B27] Jackson M. A. Verdi S. Maxan M. E. Shin C. M. Zierer J. Bowyer R. C. E. (2018). Gut microbiota associations with common diseases and prescription medications in a population-based cohort. *Nat. Commun.* 9:2655. 10.1038/s41467-018-05184-7 29985401 PMC6037668

[B28] Jiang H. Ling Z. Zhang Y. Mao H. Ma Z. Yin Y. (2015). Altered fecal microbiota composition in patients with major depressive disorder. *Brain Behav. Immun.* 48 186–194. 10.1016/j.bbi.2015.03.016 25882912

[B29] Johnston K. M. Powell L. C. Anderson I. M. Szabo S. Cline S. (2019). The burden of treatment-resistant depression: a systematic review of the economic and quality of life literature. *J. Affect. Disord.* 242 195–210. 10.1016/j.jad.2018.06.045 30195173

[B30] Jourova L. Anzenbacher P. Anzenbacherova E. (2016). Human gut microbiota plays a role in the metabolism of drugs. *Biomed. Pap. Med. Fac. Univ. Palacky Olomouc Czech. Repub.* 38 317–326. 10.5507/bp.2016.039 27485182

[B31] Keller M. B. (2003). Past, present, and future directions for defining optimal treatment outcome in depression: remission and beyond. *JAMA* 289 3152–3160. 10.1001/jama.289.23.3152 12813121

[B32] Kelly J. R. Borre Y. O’Brien C. Patterson E. El Aidy S. Deane J. (2016). Transferring the blues: depression-associated gut microbiota induces neurobehavioural changes in the rat. *J. Psychiatr. Res.* 82 109–118. 10.1016/j.jpsychires.2016.07.019 27491067

[B33] Kessler R. C. Berglund P. Demler O. Jin R. Koretz D. Merikangas K. R. (2003). The epidemiology of major depressive disorder: results from the National Comorbidity Survey Replication (NCS-R). *JAMA* 289 3095–3105. 10.1001/jama.289.23.3095 12813115

[B34] Klünemann M. Andrejev S. Blasche S. Mateus A. Phapale P. Devendran S. (2021). Bioaccumulation of therapeutic drugs by human gut bacteria. *Nature* 597 533–538. 10.1038/s41586-021-03891-8 34497420 PMC7614428

[B35] Kuczynski J. Stombaugh J. Walters W. A. González A. Caporaso J. G. Knight R. (2012). Using QIIME to analyze 16S rRNA gene sequences from microbial communities. *Curr. Protoc. Microbiol.* Chapter 1 Unit–1E.5. 10.1002/9780471729259.mc01e05s27 23184592 PMC4477843

[B36] Lai W. T. Deng W. F. Xu S. X. Zhao J. Xu D. Liu Y. H. (2021). Shotgun metagenomics reveals both taxonomic and tryptophan pathway differences of gut microbiota in major depressive disorder patients. *Psychol. Med.* 51 90–101. 10.1017/S0033291719003027 31685046

[B37] Lépine J. P. Briley M. (2011). The increasing burden of depression. *Neuropsychiatr. Dis. Treat.* 7 3–7. 10.2147/NDT.S19617 21750622 PMC3131101

[B38] Lin P. Ding B. Feng C. Yin S. Zhang T. Qi X. (2017). *Prevotella* and *Klebsiella* proportions in fecal microbial communities are potential characteristic parameters for patients with major depressive disorder. *J. Affect. Disord.* 207 300–304. 10.1016/j.jad.2016.09.051 27741466

[B39] Liu Y. Zhang L. Wang X. Wang Z. Zhang J. Jiang R. (2016). Similar fecal microbiota signatures in patients with diarrhea-predominant irritable bowel syndrome and patients with depression. *Clin. Gastroenterol. Hepatol.* 14 1602–1611.e5. 10.1016/j.cgh.2016.05.033 27266978

[B40] Long-Smith C. O’Riordan K. J. Clarke G. Stanton C. Dinan T. G. Cryan J. F. (2020). Microbiota-gut-brain axis: new therapeutic opportunities. *Annu. Rev. Pharmacol. Toxicol.* 60 477–502. 10.1146/annurev-pharmtox-010919-023628 31506009

[B41] Maalouf F. T. Brent D. A. (2012). Child and adolescent depression intervention overview: what works, for whom and how well? *Child Adolesc. Psychiatr. Clin. N. Am.* 21 299–312. 10.1016/j.chc.2012.01.001 22537728

[B42] Macedo D. Filho A. J. M. C. Soares de Sousa C. N. Quevedo J. Barichello T. Júnior H. V. N. (2017). Antidepressants, antimicrobials or both? gut microbiota dysbiosis in depression and possible implications of the antimicrobial effects of antidepressant drugs for antidepressant effectiveness. *J. Affect. Disord.* 208 22–32. 10.1016/j.jad.2016.09.012 27744123

[B43] Maes M. Bosmans E. De Jongh R. Kenis G. Vandoolaeghe E. Neels H. (1997). Increased serum IL-6 and IL-1 receptor antagonist concentrations in major depression and treatment resistant depression. *Cytokine* 9 853–858. 10.1006/cyto.1997.0238 9367546

[B44] Manchia M. Pisanu C. Squassina A. Carpiniello B. (2020). Challenges and future prospects of precision medicine in psychiatry. *Pharmgenom. Pers. Med.* 13 127–140. 10.2147/PGPM.S198225 32425581 PMC7186890

[B45] Mason B. L. Li Q. Minhajuddin A. Czysz A. H. Coughlin L. A. Hussain S. K. (2020). Reduced anti-inflammatory gut microbiota are associated with depression and anhedonia. *J. Affect. Disord.* 266 394–401. 10.1016/j.jad.2020.01.137 32056905

[B46] Mayer E. A. Knight R. Mazmanian S. K. Cryan J. F. Tillisch K. (2014). Gut microbes and the brain: paradigm shift in neuroscience. *J. Neurosci.* 34 15490–15496. 10.1523/JNEUROSCI.3299-14.2014 25392516 PMC4228144

[B47] McGuinness A. J. Davis J. A. Dawson S. L. Loughman A. Collier F. O’Hely M. (2022). A systematic review of gut microbiota composition in observational studies of major depressive disorder, bipolar disorder and schizophrenia. *Mol. Psychiatry* 27 1920–1935. 10.1038/s41380-022-01456-3 35194166 PMC9126816

[B48] Munoz-Bellido J. L. Munoz-Criado S. Garcìa-Rodrìguez J. A. (2000). Antimicrobial activity of psychotropic drugs: selective serotonin reuptake inhibitors. *Int. J. Antimicrob. Agents* 14 177–180. 10773485 10.1016/s0924-8579(99)00154-5

[B49] Olesen O. V. Linnet K. (1996). Simplified high-performance liquid chromatographic method for the determination of citalopram and desmethylcitalopram in serum without interference from commonly used psychotropic drugs and their metabolites. *J. Chromatogr. B Biomed. Appl.* 675 83–88. 10.1016/0378-4347(95)00347-9 8634772

[B50] Pisanu C. Squassina A. (2017). We are not alone in our body: insights into the involvement of microbiota in the etiopathogenesis and pharmacology of mental illness. *Curr. Drug Metab.* 19 688–694. 10.2174/1389200219666171227204144 29283065

[B51] Qu Y. Yang C. Qian R. Min M. Chao D. Hashimoto K. (2017). Comparison of (R)-ketamine and lanicemine on depression-like phenotype and abnormal composition of gut microbiota in a social defeat stress model. *Sci. Rep.* 7:15725. 10.1038/s41598-017-16060-7 29147024 PMC5691133

[B52] Rong H. Xie X. H. Zhao J. Lai W. T. Wang M. B. Xu D. (2019). Similarly in depression, nuances of gut microbiota: evidences from a shotgun metagenomics sequencing study on major depressive disorder versus bipolar disorder with current major depressive episode patients. *J. Psychiatr. Res.* 113 90–99. 10.1016/j.jpsychires.2019.03.017 30927646

[B53] Sanada K. Nakajima S. Kurokawa S. Barceló-Soler A. Ikuse D. Hirata A. (2020). Gut microbiota and major depressive disorder: a systematic review and meta-analysis. *J. Affect. Disord.* 266 1–13. 10.1016/j.jad.2020.01.102 32056863

[B54] Schwarz E. Maukonen J. Hyytiäinen T. Kieseppä T. Orešič M. Sabunciyan S. (2018). Analysis of microbiota in first episode psychosis identifies preliminary associations with symptom severity and treatment response. *Schizophr. Res.* 192 398–403. 10.1016/j.schres.2017.04.017 28442250

[B55] Shen Y. Yang X. Li G. Gao J. Liang Y. (2021). The change of gut microbiota in MDD patients under SSRIs treatment. *Sci. Rep.* 11:14918. 10.1038/s41598-021-94481-1 34290352 PMC8295378

[B56] Simpson C. A. Diaz-Arteche C. Eliby D. Schwartz O. S. Simmons J. G. Cowan C. S. M. (2021). The gut microbiota in anxiety and depression–a systematic review. *Clin. Psychol. Rev.* 83:101943. 10.1016/j.cpr.2020.101943 33271426

[B57] Siopi E. Chevalier G. Katsimpardi L. Saha S. Bigot M. Moigneu C. (2020). Changes in gut microbiota by chronic stress impair the efficacy of fluoxetine. *Cell Rep.* 30 3682–3690.e6. 10.1016/j.celrep.2020.02.099 32187541

[B58] Thomas S. J. Shin M. McInnis M. G. Bostwick J. R. (2015). Combination therapy with monoamine oxidase inhibitors and other antidepressants or stimulants: strategies for the management of treatment-resistant depression. *Pharmacotherapy* 35 433–449. 10.1002/phar.1576 25884531

[B59] Valles-Colomer M. Falony G. Darzi Y. Tigchelaar E. F. Wang J. Tito R. Y. (2019). The neuroactive potential of the human gut microbiota in quality of life and depression. *Nat. Microbiol.* 4 623–632. 10.1038/s41564-018-0337-x 30718848

[B60] Vich Vila A. Collij V. Sanna S. Sinha T. Imhann F. Bourgonje A. R. (2020). Impact of commonly used drugs on the composition and metabolic function of the gut microbiota. *Nat. Commun.* 11:362. 10.1038/s41467-019-14177-z 31953381 PMC6969170

[B61] Vinberg M. Ottesen N. M. Meluken I. Sørensen N. Pedersen O. Kessing L. V. (2019). Remitted affective disorders and high familial risk of affective disorders associate with aberrant intestinal microbiota. *Acta Psychiatr. Scand.* 139 174–184. 10.1111/acps.12976 30374951

[B62] Vivarelli S. Salemi R. Candido S. Falzone L. Santagati M. Stefani S. (2019). Gut microbiota and cancer: from pathogenesis to therapy. *Cancers* 11:38. 10.3390/cancers11010038 30609850 PMC6356461

[B63] Yamamura R. Okubo R. Katsumata N. Odamaki T. Hashimoto N. Kusumi I. (2021). Lipid and energy metabolism of the gut microbiota is associated with the response to probiotic *Bifidobacterium breve* strain for anxiety and depressive symptoms in schizophrenia. *J. Pers. Med.* 11:987. 10.3390/jpm11100987 34683128 PMC8539730

[B64] Yang C. Qu Y. Fujita Y. Qian R. Min M. Chao D. (2017). Possible role of the gut microbiota–brain axis in the antidepressant effects of (R)-ketamine in a social defeat stress model. *Transl. Psychiatry* 7:1294. 10.1038/s41398-017-0031-4 29249803 PMC5802627

[B65] Ye X. Wang D. Zhu H. Wang D. Li J. Tang Y. (2021). Gut microbiota changes in patients with major depressive disorder treated with vortioxetine. *Front. Psychiatry* 12:641491. 10.3389/fpsyt.2021.641491 34025474 PMC8138160

[B66] Yilmaz P. Parfrey L. W. Yarza P. Gerken J. Pruesse E. Quast C. (2014). The SILVA and “all-species Living Tree Project (LTP)” taxonomic frameworks. *Nucleic Acids Res.* 42 D643–D648. 10.1093/nar/gkt1209 24293649 PMC3965112

[B67] Yong S. J. Tong T. Chew J. Lim W. L. (2019). Antidepressive mechanisms of probiotics and their therapeutic potential. *Front. Neurosci.* 13:1361. 10.3389/fnins.2019.01361 32009871 PMC6971226

[B68] Zhang J. Zhang J. Wang R. (2018). Gut microbiota modulates drug pharmacokinetics. *Drug Metab. Rev.* 50 1–12. 10.1080/03602532.2018.1497647 30227749

[B69] Zhang W. Qu W. Wang H. Yan H. (2021). Antidepressants fluoxetine and amitriptyline induce alterations in intestinal microbiota and gut microbiome function in rats exposed to chronic unpredictable mild stress. *Transl. Psychiatry* 11:131. 10.1038/s41398-021-01254-5 33602895 PMC7892574

[B70] Zhao K. Li W. Kang C. Du L. Huang T. Zhang X. (2014). Phylogenomics and evolutionary dynamics of the family Actinomycetaceae. *Genome Biol. Evol.* 6 2625–2633. 10.1093/gbe/evu211 25245410 PMC4224338

[B71] Zheng P. Zeng B. Zhou C. Liu M. Fang Z. Xu X. (2016). Gut microbiome remodeling induces depressive-like behaviors through a pathway mediated by the host’s metabolism. *Mol. Psychiatry* 21 786–796. 10.1038/mp.2016.44 27067014

[B72] Zhernakova A. Kurilshikov A. Bonder M. J. Tigchelaar E. F. Schirmer M. Vatanen T. (2016). Population-based metagenomics analysis reveals markers for gut microbiome composition and diversity. *Science* 352 565–569. 10.1126/science.aad3369 27126040 PMC5240844

